# Stereotactic Body Radiation Therapy for Symptomatic Pancreatic Insulinoma: Two-Case Report and Literature Review

**DOI:** 10.3390/curroncol31070307

**Published:** 2024-07-22

**Authors:** Agnieszka Namysl-Kaletka, Jerzy Wydmanski, Iwona Debosz-Suwinska, Malgorzata Kaszuba, Dorota Gabrys, Agata Roch-Zniszczol, Daria Handkiewicz-Junak

**Affiliations:** 1 Radiotherapy Department, Maria Sklodowska-Curie National Research Institute of Oncology, Gliwice Branch, 44-102 Gliwice, Poland; 2 Radiology and Diagnostic Imaging Department, Maria Sklodowska-Curie National Research Institute of Oncology, Gliwice Branch, 44-102 Gliwice, Poland; 3 Department of Nuclear Medicine and Endocrine Oncology, Maria Sklodowska-Curie National Research Institute of Oncology, Gliwice Branch, 44-102 Gliwice, Poland

**Keywords:** pancreatic insulinoma, stereotactic body radiation therapy, local control

## Abstract

Insulinoma is the most common functional neuroendocrine tumor of the pancreas, with the main clinical symptom being hypoglycemia. The standard treatment is surgery, but some patients are not eligible for surgery, while in those operated on, the risk of perioperative complications is up to 30%. Diazoxide treatment to prevent hypoglycemia is effective only in 50% of patients. To prevent tumor growth and hormonal excess, stereotactic radiotherapy may be an alternative to surgical treatment. In our paper, we present two cases of patients with insulinoma treated successfully with stereotactic body radiation therapy (SBRT).

## 1. Introduction

About 50% of well-differentiated neuroendocrines are functional tumors, and insulinoma, with a frequency of one to three cases per million people per year, is the most frequent [1]. It originates from the B cells of the pancreatic islets and produces insulin, so the diagnosis is prompted by symptoms of hypoglycemia, sometimes leading to neuroglycopenic symptoms (confusion, seizures, blurred vision, coma). In 5–10% of cases, insulinoma is malignant with local infiltration and metastasis to the lymph nodes and liver [2]. With modern imaging tools, the location of insulinoma can be adequately achieved before surgery with varying sensitivity: transabdominal ultrasound ranging from 9% to 64% [3], CT 33 to 64%, MRI 40 to 90% [4], and endoscopic ultrasound—EUS 57–94% [5]. A parenchymal-sparing pancreatectomy should be proposed as the first-line surgical strategy [6]; however, perioperative mortality ranges from 6 to 10% [7,8,9]. Due to the risk of perioperative complications, lack of consent for surgery, or diagnosis of a locally nonresectable tumor, some patients are ineligible for surgical treatment. In that case, conservative interventional radioembolization can be an option. Liver-directed radioembolization with chemoembolization was found to be highly effective in controlling hypoglycemia in a series of seven patients with metastatic malignant insulinoma of the liver, resulting in an initial clinical success rate of 100% and an overall clinical success rate of 85% [10,11]. Huscher et al. presented the first case report of the treatment of insulinoma with stereotactic radiosurgery, where they achieved 3-year clinical control of symptoms [12]. In this article, we present two patients with pancreatic insulinoma after SBRT, in whom control of clinical and biochemical symptoms was achieved.

## 2. First Case

**Figure 1:**
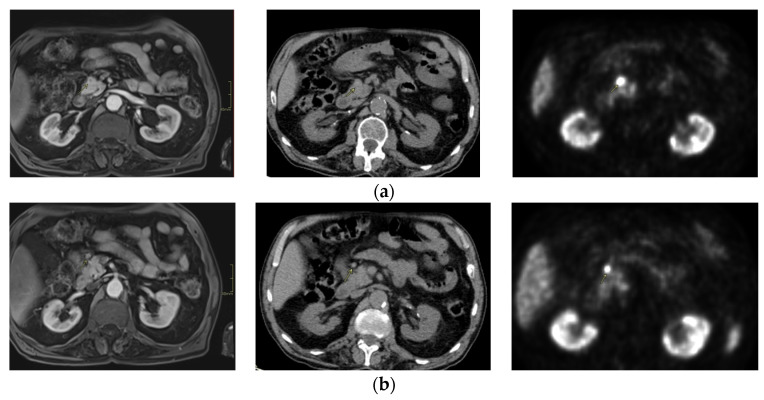
(**a**) T1 vibe Dixon MRI, CT, and PET CT of the abdomen performed in October 2018. The tumor on the abdominal part of the head of the pancreas. (**b**) T1 vibe Dixon MRI, CT, and PET CT of the abdomen performed in October 2018. Peripancreatic lymph node.

An 82-year-old man with a history of left ventricular hypertrophy, colonic diverticulosis, lumbar spine scoliosis, and stage 3 chronic renal failure was admitted to the Department of Internal Medicine due to low blood glucose concentration (2.7 mmol/L) with impaired consciousness. In November 2017, when the fasting test was performed and 2 h after the start of the test, the glucose level was 2.3 mmol/L with insulin level 12.3 ulU/mL. A CT scan of the abdomen and pelvis showed a 15 mm hypervascular lesion in the head of the pancreas. In September 2018, a PET/CT Ga68 Dotatate scan was performed, and in October, abdominal contrast enhanced magnetic resonance imaging was performed and showed a pancreatic lesion (Figure 1a) and a small (7 × 8 mm) peripancreatic lymph node (Figure 1b). Tectrotide scintigraphy was also performed. The clinical diagnosis of insulinoma was made, and the patient started treatment with diazoxide—25 mg in the morning and 100 mg in the evening. The patient was referred for pancreatic surgery; however, due to comorbidities, he did not consent to the treatment. Due to chronic renal failure, the patient was not eligible for isotope treatment or target therapy. However, because of symptomatic hypoglycemia, the daily dose of Proglicem was increased to 75–25–100 mg and the patient was referred for stereotactic radiation therapy. However, due to a sinus stroke of the left hemisphere in January 2019, stereotactic surgery was started in May 2019. Between October 2018 and May 2019, systematic treatment was adjusted to clinical symptoms, and at the start of radiotherapy, the patient was treated with Proglicem 25 mg bid and Sadostatin 120 mg sc. every 4 weeks. From 15 May to 24 May 2019, stereotactic radiation therapy was performed in the area of the pancreatic head lesion, and the lymph node located above the head of the pancreas (confirmed to express somatostatin analogues) was performed every second day with a fractional dose of 7 Gy to the total dose of 35 Gy (high-energy linear accelerator Clinac 23EX; Varian Medical Systems, Palo Alto, CA, USA). GTV was delineated on PET/CT, MR, and CT. To create PTV, a 5 mm margin to GTV was added.

**Figure 2:**
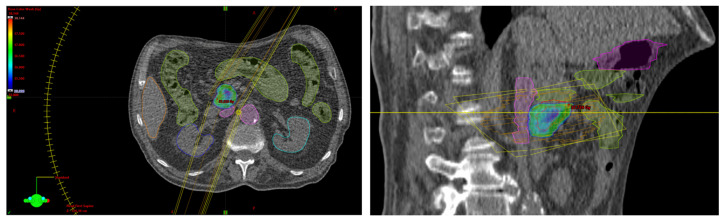
Stereotactic radiotherapy with respiratory gating at a total dose of 35 Gy in 5 fractions. The patient was treated with volumetric modulated arc therapy (VMAT). Dose distribution in the transverse and sagittal planes is shown as a dose wash color. The minimum dose was set to 35 Gy. There was a dose decrease in the proximity of the intestine (the minimum dose in PTV was 30 Gy). Colors legend: orange, liver; green, bowel; cyan, left kidney; blue, right kidney; magenta, vessels; red, GTV/PTV.

**Figure 3:**
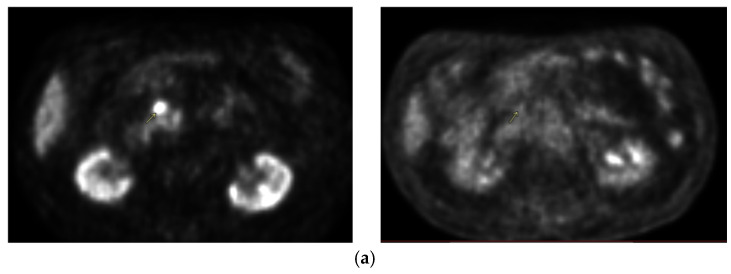

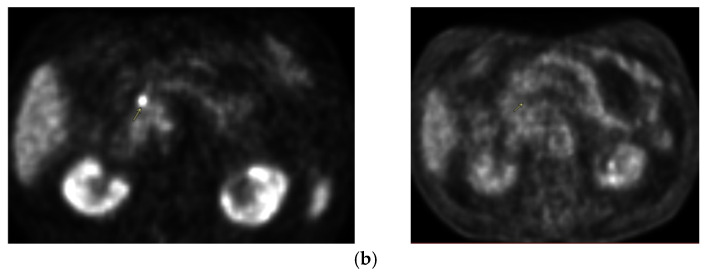
(**a**) Comparison between PET CT performed in 2018 (left scan) and November 2019 (right scan) without pathological radiotracer uptake foci corresponding to the location of the tumor lesion after SBRT. (**b**) Comparison between PET CT performed in 2018 (left scan) and November 2019 (right scan) without pathological radiotracer uptake foci corresponding to the location of the peripancreatic lymph node after SBRT.

**Figure 4:**
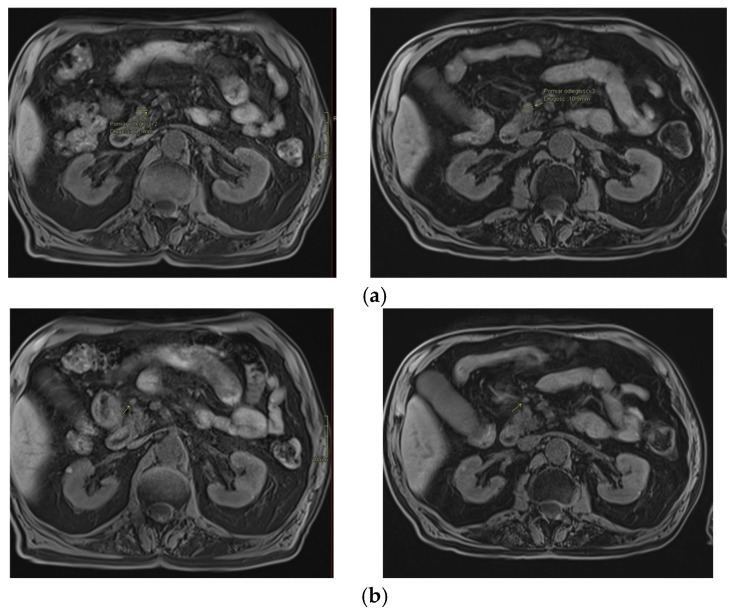
(**a**) Control MRI performed in August 2020 (left scan) and in March 2021 (right scan) of the abdomen: stable disease in the pancreatic head. (**b**) Control MRI performed in August 2020 (left scan) and in March 2021 (right scan) of the abdomen: stable disease in the adjacent lymph node.

**Figure 5:**
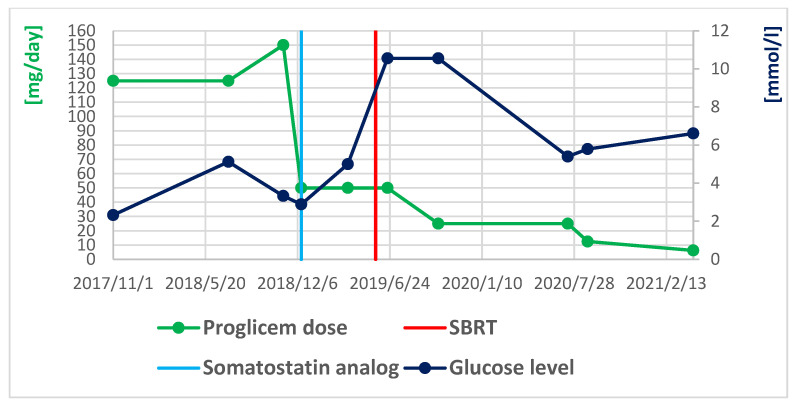
Dependency of glucose level and Proglicem dose over time in the first case.

Critical organs such as the kidney, liver, spinal canal, small intestine, large intestine, stomach, duodenum, and vessels were contoured. The total dose was 35 Gy in five fractions to limit the dose to the small intestine. The maximum dose to the small intestine was 31.73 Gy, and, according to the Timmerman’s tables, a dose of 32 Gy was recommended. Dose distribution is shown in Figure 2. Image-guided respiration-gated radiation therapy (IGRT) was used using cone beam computed tomography (CBCT). The first follow-up was 1 month after radiotherapy and after every 3 months for 6 months and every 3 to 8 months afterward. No early or late toxicity was observed during the two-year follow-up. Systemic treatment with 50 mg/d Proglycem was continued. Blood glucose and insulin were within the normal range and there were no clinical symptoms of hypoglycemia. In October 2019, a blood glucose concentration with a tendency to hyperglycemia was observed; therefore, the dose of Proglicem was reduced to 25 mg/d. In November 2019, PET/CT examination of the somatostatin receptor was performed and there was no pathological uptake corresponding to the location of the tumor lesion and peripancreatic lymph node (Figure 3). Performed in August 2020 and in March 2021, control MRI of the abdomen showed a stable disease in the head (Figure 4a) and the adjacent lymph node (Figure 4b). In the two-year follow-up, hormonal hyperactivity was not found. Changes in glucose blood concentration and Proglicem dose over time are shown in Figure 5. The patient died in May 2021 from concomitant diseases without pancreatic neuroendocrine tumor progression.

## 3. Second Case

**Figure 6:**
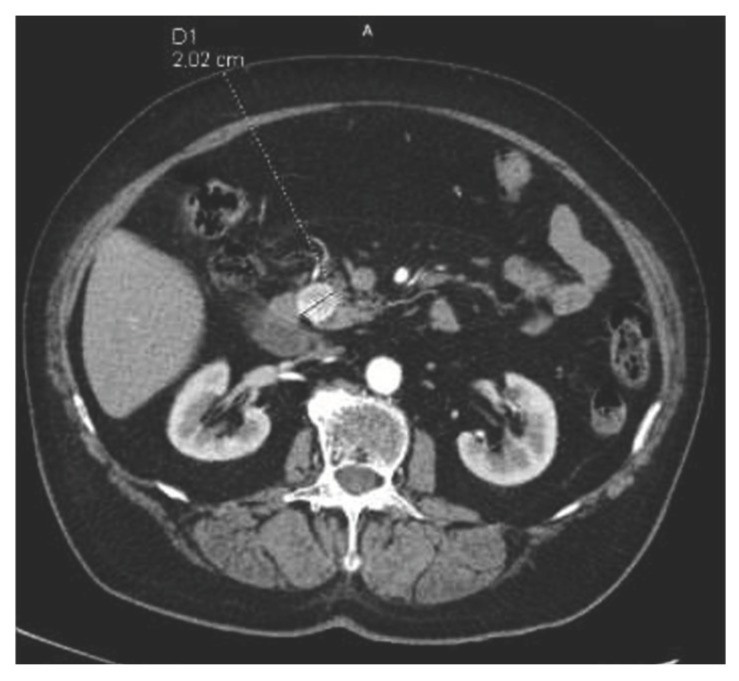
CT performed in June 2016: 20 mm tumor in pancreatic head.

**Figure 7:**
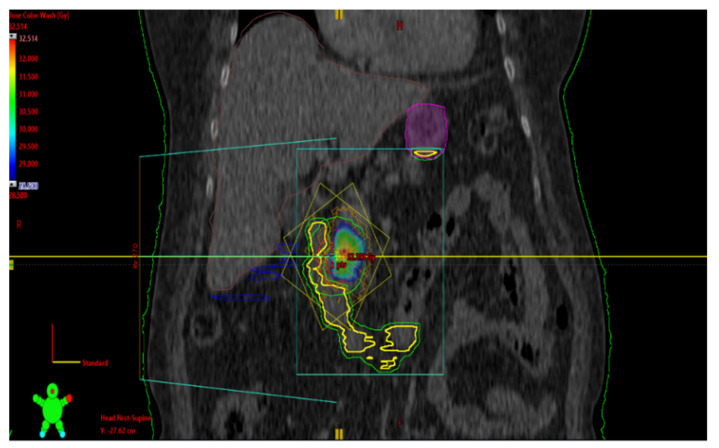
Second case. The patient was treated with VMAT. Dose distribution in the coronal plane. Isodose tumor coverage of 95% of the prescribed dose while limiting the dose to critical organs such as the duodenum. The dose is shown as a wash of colors. The minimum dose is set at 28.5 Gy. Dose decrease in OAR (organ at risk) volume, such as intestines, duodenum, spinal cord, liver, and stomach, in line with international guidelines for doses to these organs. Colors legend: yellow/green, duodenum; blue, bowel; magenta, stomach; red, GTV/CTV/PTV.

**Figure 8:**
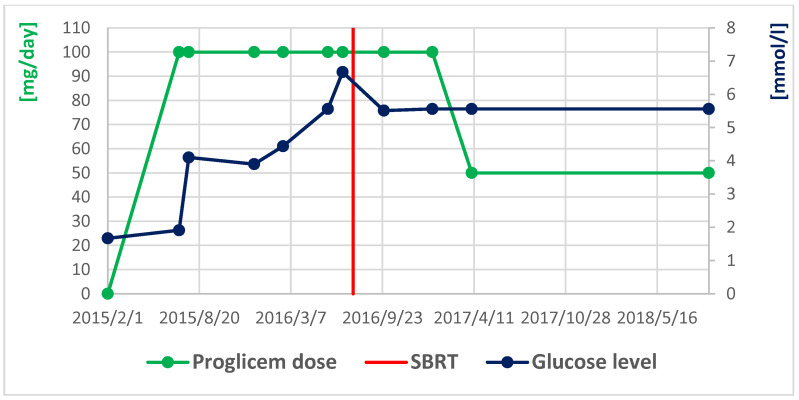
Dependency of glucose level and Proglicem dose over time in the second case.

**Figure 9:**
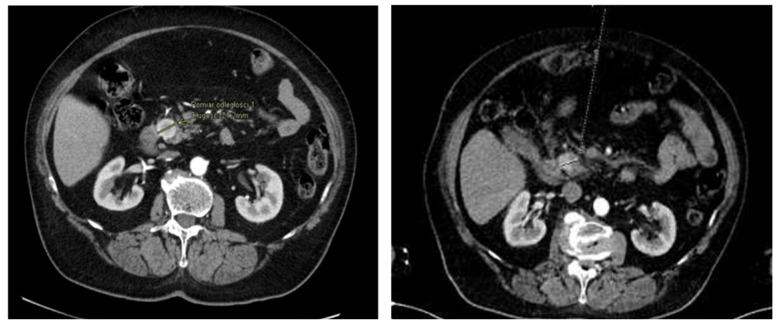
Second case. Comparison of CT performed in June 2016 (left image) and June 2017 (right image): stable tumor dimensions with a decrease in the level of contrast enhancement in the left half of the pancreatic tumor after SBRT.

An 84-year-old female with dementia was referred to the National Institute of Oncology due to the incidence of hypoglycemia (1.67 mmol/L) with temporary loss of consciousness. The patient had a history of radiotherapy for chemodectoma of the middle ear on the left side 36 years earlier and had been treated for diabetes for 10 years (the patient had not been treated pharmacologically for 9 years). In June 2016, a CT scan revealed a 20 mm tumor in the pancreatic head (Figure 6). Somatostatin receptor scintigraphy (Tc99m-tectreotide) confirmed radiotracer uptake in pancreatic head lesion. During hospitalization at the Department of Nuclear Medicine and Endocrine Oncology, the pathognomonic for the diagnosis of insulinoma, hypoglycemia (1.97 mmol/L) with inadequately high levels of insulin (24 uIU/mL) and peptide (3.14 ng/mL), was found. The diagnosis of insulinoma was made and Diazoxide was started at a dose of 100 mg/day with good biochemical (glycemia was 3.3–5.6 mmol/L) and clinical control. The patient was referred for surgery, but did not consent to it, so radiotherapy was offered to the patient. In July 2016, the patient received image-guided respiration-gated radiation therapy to the pancreatic tumor with a fractional dose of 10 Gy to a total dose of 30 Gy (Figure 7) to limit the dose to the duodenum. GTV was determined based on CT and MRI. IGRT was performed using kV images; therefore, additional margins were used: CTV-GTV margins of 5 mm and PTV-CTV of 4 mm were added. The first follow-up was 1 month after SBRT, during the next year and half every 3 months, and the last visit was after one year due to a compression fracture of the L1 vertebral body due to osteoporosis and the inability to return for a follow-up visit. In April 2017, the dose of diazoxide was reduced by 50%, that is, 50 mg once a day (Figure 8). In June 2017, a control abdominal cavity CT was performed, and stable tumor dimensions were found with a decrease in contrast enhancement in the left half of the tumor (Figure 9). During follow-up, no early or late side effects were diagnosed.

The patient died in March 2019 due to comorbidities and deterioration of the general condition without symptoms of radiological or biochemical progression.

## 4. Discussion

Diagnosis of insulinoma is based on clinical picture and consistent biochemical tests, and low glucose level (2.5 mmol/l) accompanied by high insulin level (more than 6 μU/mL) [10]. Other biochemical markers include elevated levels of C-peptide, proinsulin, and β-hydroxybutyrate. Factitious hypoglycemia should be ruled out with assessment of oral hypoglycemic agents [13]. Preoperative location of tumors as small as 5 mm can often be achieved using endoscopic ultrasound, CT, and/or magnetic resonance imaging [14]. Wei et al. reported that the detection rates for transabdominal ultrasound, CT, MRI, and EUS are 22%, 72%, 75%, and 80%, respectively [15]. EUS in combination with fine needle aspiration biopsy (FNA) achieves sensitivity and specificity above 90% [16,17], although somatostatin receptor imaging (SRI) is a gold standard in diagnostic work-up, and well-differentiated neuroendocrine tumors in insulinoma A meta-analysis based on 18 publications showed sensitivity and specificity of SRI in patients with PanNET at 79.6% (95% CI: 71–87%) and 95% (95% CI: 75–100%), appropriately [18]. Some data suggest that insulinomas express highly glucagon-like peptide-1 receptors (GLP-1R) and, thus, are labeled with a GLP-1R agonist [19]. When the diagnosis of insulinoma is confirmed, one of the most important treatment goals is the prevention of severe hypoglycemia. Diazoxide, a potassium channel opener on β-pancreatic islet, which decreases insulin secretion, is a treatment of choice [20]. However, diazoxide is effective in only about 50% of cases [21] and may cause a number of side effects, such as peripheral oedema, congestive heart failure, renal failure, hypotension, weight gain, and excessive hair. The effectiveness of somatostatin analogues in preventing hypoglycemic episodes is estimated at 40–60% of cases, but it can inhibit the release of counterregulatory hormones, such as glucagon, which can even worsen hypoglycemia in some patients [22]. Due to symptoms, surgical treatment is recommended. In patients eligible for surgery, tumor enucleation or more extensive resection as anatomically required is the optimal radical treatment, and a lymph node or nodes should be collected during the procedure for diagnostic assessment [5]. In this group of patients, EUS radiofrequency ablation remains an alternative treatment method, especially for tumors smaller than 2 cm [23]. Also, EUS-directed ablation using ethanol injection or CT-guided RFA appears to be a promising approach [24]. Its main advantage is low rate of serious side effects compared with surgery. In a systematic review and meta-analysis, the overall AE rates for clinical efficacy were 17.8% and 95.1% for functional pancreatic neuroendocrine tumors treated with endoscopic ultrasound-guided radiofrequency ablation [25]. Currently, enucleation is performed in 56% of patients, distal pancreatectomy in 32%, Whipple in 3%, and subtotal pancreatectomy in less than 3%, while 0.5% of cases undergo exploratory laparotomy and biopsy. However, surgical treatment is associated with the risk of complications. Even with laparoscopic treatment, pancreatic fistula (7.2%), abscess (2%), diabetes mellitus (7.5%), pancreatitis (3.1%), and pulmonary embolism (1.8%) are observed. SBRT is not an invasive method that, as in our cases, can be safely applied in operable patients with major comorbidities to achieve stabilization of the disease with a decrease in drug intake [26]. SBRT as a noninvasive therapy allows for administration of high doses to a small tissue volume to control or destroy tumor cells, which allows for sparing organs. In both cases, we draw critical organs such as the kidney, liver, spinal canal, small intestine, large intestine, stomach and duodenum, and vessels. When the patients described in the publication were irradiated, we used the dose recommendations from the available Timmerman’s tables. We are currently relying on the updated information from Timmerman’s published recommendations [27]. The GTV was delineated on MR, CT, and PET/CT in the first case. The added margin to the GTV depended on the type of verification before fraction-gated CBCT/kV. The total dose depended on the location of the critical organs. SBRT does not require hospitalization and the patient receives 1–5 fractions of treatment. The treatment itself generally lasts from several minutes to an hour. The benefits for patients not qualified for surgical treatment due to concomitant diseases are the lack of general anesthesia, painlessness, and no need to modify the current pharmacological treatment.

In 2012, Huscher described the first case of insulinoma successfully treated with stereotactic radiotherapy, with a follow-up of 3 years [12]. The patient received 25 Gy in a single dose using the Cyber-Knife. Similarly, Abdulrahman Alsuhaibani et al. described a case of a patient who underwent stereotactic radiation therapy with a multifractional scheme of 40 Gy in four fractions. The authors reported long-lasting biochemical and clinical improvement in hypoglycemia incidents and lowered serum insulin levels, and did not observe side effects at the five-month follow-up [28]. The optimal dose fractionation is a crucial issue in neuroendocrine tumors, since life expectancy in this group of patients is relatively long. We use multifraction regimens in our patients due to concerns about gastrointestinal toxicity. Schellenberg et al. showed that the use of a single dose of 25 Gy in locally advanced pancreatic cancer resulted in an increased risk of duodenal ulcers, strictures, and perforation [29]. Pollom et al. compared single- versus multifraction stereotactic body radiation therapy for pancreatic adenocarcinoma and there were significantly fewer instances of toxicity grade ≥ 2 with multifraction SBRT [30]. To decrease radiation to normal tissues, we used respiratory gating and verification based on cone beam computer tomography (CBCT) or kV imaging before each fraction of radiotherapy. In the case of our patients, we did not observe any radiation-induced side effects. Treatment was well tolerated. No grade 3 or 4 toxicity was observed in the cited articles and our experiments. During the follow-up period of 2 years in the first case and 2 years and 9 months in the second case, there was no radiological or biochemical progression of the disease.

Irradiation with high doses per fraction induces indirect death of tumor cells. Such indirect cell death after high-dose radiation is likely due to radiation-induced vascular damage and the ensuing deterioration of the intratumor microenvironment [31]. In addition, the immune response for local ablative treatment is complex, including both immunostimulatory and suppressive elements [32].

There are a lot of data in the literature on the pharmacological and surgical treatment of pancreatic insulinoma, but descriptions of individual cases treated with stereotactic radiotherapy are very scarce. Both the cited publications and our experience show that stereotactic radiation therapy is a safe and effective alternative to surgical treatment for patients who do not consent to such treatment or who do not qualify for it due to concomitant diseases.

In preclinical models, L19-IL2 has been shown to enhance local and abscopal effects of radiation therapy [33]; perhaps the combination of systemic treatment with SBRT can increase the chances of complete response and resolution of clinical symptoms.

Due to the risk of perioperative complications reaching even 30%, stereotactic radiation therapy may become the first-line treatment of choice. However, due to the small number of publications, a randomized multicenter clinical trial is indicated.

## 5. Conclusions

Stereotactic radiotherapy may be an effective and well-tolerated method for treating symptomatic, inoperable pancreatic insulinomas.

## Data Availability

Data are contained within the article.

## References

[1] Okabayashi T., Shima Y., Sumiyoshi T., Kozuki A., Ito S., Ogawa Y., Kobayashi M., Hanazaki K. (2013). Diagnosis and management of insulinoma. World J. Gastroenterol..

[2] De Herder W.W., Niederle B., Scoazec J.Y., Pauwels S., Klöppel G., Falconi M., Kwekkeboom D.J., Öberg K., Eriksson B., Wiedenmann B. (2006). Diego FeroneFrascati Consensus Conference; European Neuroendocrine Tumor Society: Well-differentiated pancreatic tumor/carcinoma: Insulinoma. Neuroendocrinology.

[3] Tucker O.N., Crotty P.L., Conlon K.C. (2006). The management of insulinoma. Br. J. Surg..

[4] McAuley G., Delaney H., Colville J., Lyburn I., Worsley D., Govender P., Torreggiani W.C. (2005). Multimodality preoperative imaging of pancreatic insulinomas. Clin. Radiol..

[5] Kos-Kudła B., Rosiek V., Borowska M., Bednarczuk T., Bolanowski M., Chmielik E., Ćwikła J.B., Foltyn W., Gisterek I., Handkiewicz-Junak D. (2022). Pancreatic neuroendocrine neoplasms—Update of the diagnostic and therapeutic guidelines (recommended by the Polish Network of Neuroendocrine Tumours) [Nowotwory neuroendokrynne trzustki—Uaktualnione zasady diagnostyki i leczenia (rekomendowane przez Polską Sieć Guzów Neuroendokrynych)]. Endokrynol. Pol..

[6] De Carbonnières A., Challine A., Cottereau A.S., Coriat R., Soyer P., Abou Ali E., Prat F., Terris B., Bertherat J., Dousset B. (2021). Surgical management of insulinoma over three decades. HPB.

[7] Swanson R.S., Pezzi C.M., Mallin K., Loomis A.M., Winchester D.P. (2014). The 90-day mortality after pancreatectomy for cancer is double the 30- day mortality: More than 20,000 resections from the national cancer data base. Ann. Surg. Oncol..

[8] El Amrani M., Clément G., Lenne X., Laueriere C., Turpin A., Theis D., Pruvot F.R., Truant S. (2020). Should all pancreatic surgery be centralized regardless of patients’ comorbidity?. HPB.

[9] El Amrani M., Lenne X., Clement G., Delpero J.R., Theis D., Pruvot F.R., Bruandet A., Truant S. (2019). Specificity of procedure volume and its association with post operative mortality in digestive cancer surgery: A nationwide study of 225,752 patients. Ann. Surg..

[10] Hofland J., Falconi M., Christ E., Castaño J.P., Faggiano A., Lamarca A., Perren A., Petrucci S., Prasad V., Ruszniewski P. (2023). European Neuroendocrine Tumor Society 2023 guidance paper for functioning pancreatic neuroendocrine tumour syndromes. J. Neuroendocrinol..

[11] Habibollahi P., Bai H.X., Sanampudi S., Soulen M.C., Dagli M. (2020). Effectiveness of Liver-Directed Therapy for the Management of Intractable Hypoglycemia in Metastatic Insulinoma. Pancreas.

[12] Huscher C.G.S., Mingoli A., Sgarzini G., Mereu A., Gasperi M. (2012). Image-guided robotic radiosurgery (Cyber Knife) for pancreatic insulinoma: Is laparoscopy becoming old?. Surg. Innov..

[13] Giannis D., Moris D., Karachaliou G.S., Tsilimigras D.I., Karaolanis G., Papalampros A., Felekouras E. (2020). Insulinomas: From diagnosis to treatment. A review of the literature. J. BUON.

[14] Ehehalt F., Saeger H.D., Schmidt C.M., Grützmann R. (2009). Neuroendocrine tumors of the pancreas. Oncologist.

[15] Wei J., Liu X., Wu J., Xu W., Gao W., Jiang K., Zhang Z., Miao Y. (2016). Diagnosis and surgical management of insulinomas in 33 consecutive patients at a single institution. Langenbecks Arch. Surg..

[16] Atiq M., Bhutani M.S., Bektas M., Lee J.E., Gong Y., Tamm E.P., Shah C.P., Ross W.A., Yao J., Raju G.S. (2012). EUS-FNA for pancreatic neuroendocrine tumors: A tertiary cancer center experience. Dig. Dis. Sci..

[17] Zografos G.N., Stathopoulou A., Mitropapas G., Karoubalis J., Kontogeorgos G., Kaltsas G., Piaditis G., Papastratis G.I. (2005). Preoperative imaging and localization of small sized insulinoma with EUS-guided fine needle tattoing: A case report. Hormones.

[18] Calabrò D., Argalia G., Ambrosini V. (2020). Role of PET/CT and Therapy Management of Pancreatic Neuroendocrine Tumors. Diagnostics.

[19] Antwi K., Fani M., Heye T., Nicolas G., Rottenburger C., Kaul F., Merkle E., Zech C.J., Boll D., Vogt D.R. (2018). Comparison of glucagon-like peptide-1 receptor (GLP-1R) PET/CT, SPECT/CT and 3T MRI for the localisation of occult insulinomas: Evaluation of diagnostic accuracy in a prospective crossover imaging study. Eur. J. Nucl. Med. Mol. Imaging.

[20] Gill G.V., Rauf O., McFarlane I.A. (1997). Diazoxide treatment for insulinoma: A national UK survey. Postgrad. Med. J..

[21] Niitsu Y., Minami I., Izumiyama H., Hashimoto K., Yoshimoto T., Satou F., Tsujino M., Ota K., Kudo A., Tanabe M. (2019). Clinical outcomes of 20 Japanese patients with insulinoma treated with diazoxide. Endocr. J..

[22] Ito T., Jensen R.T. (2021). Perspectives on the current pharmacotherapeutic strategies for management of functional neuroendocrine tumor syndromes. Expert Opin. Pharmacother..

[23] Rimbaş M., Rizzatti G., Tosoni A., Impagnatiello M., Panzuto F., Larghi A. (2022). Small nonfunctional pancreatic neuroendocrine neoplasms: Time for a step-up treatment approach?. Endosc. Ultrasound..

[24] Falconi M., Eriksson B., Kaltsas G., Bartsch D.K., Capdevila J., Caplin M., Kos-Kudla B., Kwekkeboom D., Rindi G., Klöppel G. (2016). Vienna Consensus Conference participants. ENETS Consensus Guidelines Update for the Management of Patients with Functional Pancreatic Neuroendocrine Tumors and Non-Functional Pancreatic Neuroendocrine Tumors. Neuroendocrinology.

[25] Armellini E., Facciorusso A., Crinò S.F. (2023). Efficacy and Safety of Endoscopic Ultrasound-Guided Radiofrequency Ablation for Pancreatic Neuroendocrine Tumors: A Systematic Review and Metanalysis. Medicina.

[26] Mehrabi A., Fischer L., Hafezi M., Dirlewanger A., Grenacher L., Diener M.K., Fonouni H., Golriz M., Garoussi C., Fard N. (2014). A systematic review of localization, surgical treatment options, and outcome of insulinoma. Pancreas.

[27] Timmerman R. (2022). A Story of Hypofractionation and the Table on the Wall. Int. J. Radiat. Oncol. Biol. Phys..

[28] Alsuhaibani A.A., Alsuhaibani A.A., Hassan T.S. (2022). Curative treatment of pancreatic functioning insulinoma with stereotactic ablative radiation therapy: Case report. Int. Surg. J..

[29] Schellenberg D., Goodman K.A., Lee F., Chang S., Kuo T., Ford J.M., Fisher G.A., Quon A., Desser T.S., Norton J. (2008). Gemcitabine chemotherapy and single-fraction stereotactic body radiotherapy for locally advanced pancreatic cancer. Int. J. Radiat. Oncol. Biol. Phys..

[30] Pollom E.L., Alagappan M., von Eyben R., Kunz P.L., Fisher G.A., Ford J.A., Poultsides G.A., Visser B.C., Norton J.A., Kamaya A. (2014). Single- versus multifraction stereotactic body radiation therapy for pancreatic adenocarcinoma: Outcomes and toxicity. Int. J. Radiat. Oncol. Biol. Phys..

[31] Song C.W., Park I., Cho L.C., Yuan J., Dusenbery K.E., Griffin R.J., Levitt S.H. (2014). Is indirect cell death involved in response of tumors to stereotactic radiosurgery and stereotactic body radiation therapy?. Int. J. Radiat. Oncol. Biol. Phys..

[32] Keisari Y. (2017). Tumor abolition and antitumor immunostimulation by physico-chemical tumor ablation. Front. Biosci..

[33] Jan Van Limbergen E., Lieverse R.I.Y., Houben R., Overhof C., Postma A., Zindler J., Verhelst F., Dubois L.J., De Ruyssche D., Lambin P. (2021). Toxicity of L19-Interleukin 2 Combined with Stereotactic Body Radiation Therapy: A Phase 1 Study. Int. J. Radiat. Oncol. Biol. Phys..

